# Association of *APOA1*-75G/A and +83C/T polymorphic variation with acute coronary syndrome patients in Kashmir (India)

**DOI:** 10.34172/jcvtr.2021.09

**Published:** 2021-01-24

**Authors:** Arshad A. Pandith, Irfan Ahmad Bhat, Iqra Niyaz, Iqbal Qasim, Ina A. Bhat, Usma Manzoor, Aabid M. Koul

**Affiliations:** ^1^Advanced Center for Human Genetics, SK Institute of Medical Sciences (SKIMS), Srinagar, J & K 190011, India; ^2^Department of Medicine, Government Medical College, 190010, Srinagar, J & K, India; ^3^Department of Clinical Biochemistry, University of Kashmir, J & K-190011, India

**Keywords:** Acute Coronary Syndrome, Cardiovascular Disease, APOA1 Gene, Polymorphism, Haplotype, Kashmir

## Abstract

***Introduction:*** Acute coronary syndrome (ACS) comes under the ambit of cardiovascular disease.APOA-1 gene plays a vital role in lipid metabolism and has been observed to have plausible role in ACS. This cross sectional case-control study was conducted to evaluate association between *APOA* 1-75G/A(rs1799837), +83C/T (rs5069) genotypes and risk for ACS.

***Methods:*** The current case-control study that included confirmed 90 ACS cases and 150 healthy controls were genotyped for *APOA* 1-75 G/A and +83 C/T by Polymerase Chain Reaction-Restriction Fragment Length Polymorphism (PCR-RFLF) method.

***Results:****APOA* 1-75G/A distribution of genotypes/alleles among cases and controls was seen proportionally same with no association to ACS (*P* = 0.5). *APOA* 1+83 C/T variants showed protective effect with ACS where variant TT genotype presented more in controls (12%) than cases (1.6%) (*P* = 0.004) and likewise variant ‘T’ allele was found more in controls than ACS cases (9.4% vs.28.5% respectively: *P* < 0.05). Further, significantly high difference of CT genotype was seen among cases and controls 15% vs. 33% respectively (*P* = 0.002). The overall distribution of different haplotypes showed a marked difference in GT when compared with GC between cases and controls (*P* = 0.0001).

***Conclusion:*** The study shows that TT genotype and variant T allele of *APOA* 1 +83 C/T depicted a protective role with respect to ACS whereas *APOA* 1-75G>A showed no relation. Haplotype GT was observed to significantly over-represent in controls with its protective effect in ACS as against wild type haplotype GC.

## Introduction

Acute coronary syndrome (ACS) is one among several conditions of cardiovascular disease.^1^ ACS is believed to determine clearly the disease progression associated with myocardial ischemia and includes unstable angina and myocardial infarction (MI) under its ambit.^2^ Symptomatically ACS comprises a continuous intensity from non–ST segment elevation MI (NSTEMI) to ST-segment elevation MI (STEMI). The Indian origin from the Asian subcontinent population accounts for a fifth of all worldwide deaths from CHD that includes ACS and overall 7 million deaths occur as per the estimates each year together due to CAD and ACS.^3,4^ ACS comprising about half of the global burden is now considered to be the prime cause of deaths in the Asia-Pacific region.^4^ ACS is a multi-faceted problem that results due to genetic variations, environmental effect, dietary habits, and lifestyle. It is known that ACS often results from the compound effects of altered multiple genes where in most cases it is not clear whether ACS is inherited in a dominant or recessive manner. Currently the LDL receptor (*LDLR*) gene,^5^ *ApoB*-100 gene,^6-9^ *ARH* gene^10^ and *ABCA*1,^11,12^ have been identified as disease causing genes for familial hypercholesterolemia and Tangier disease, a risk factor for ACS.

The *APOA*1 -75 G/A and +83 C/T gene polymorphic variations is comprehensively investigated and found to be associated with other disease as well. Bi Hong et al^13^ investigated the role of *APOA*1 -75 G/A and +83 C/T SNPs and found *APOA*1-75 A allele to have a lower risk of CAD. Also, a pilot study in North India^14^ found association of *APOA*1 +83 C/T in patient with MI. In fact, it is strongly believed that above *APOA*1 polymorphic sequence variations have an important role in a host of other diseases like Alzheimer’s, breast cancer and schizophrenia etc. The genetic nature of variability accounts for nearly 50% in the plasma HDL cholesterol concentration and among many one such sequence variants is within the *APOA*1 gene, where a guanine to adenine transition occurs 75 base pairs upstream from the start of transcription (-75G/A) and other site (first intron) of the *APOA*1 gene results with transition of cytosine to thymine at +83bp (+83 C/T).Although ACS is the most prevalent disease throughout the world yet our population (Kashmir province, North India) is also heavily burdened with the same disease. The overall prevalence of coronary artery disease in our region as calculated by different diagnostic procedures stands at 7.54% where its frequency in males is higher 7.80% versus 6.63% in females.^15^ Keeping the well-recognized role of *APOA*1 gene in ACS in many populations of the world but no study till date has been done on *APOA*1 gene SNPs with respect to ACS in Kashmiri population. Thus with an increasing number of ACS cases being reported here with lack of any significant study, we designed our study with an aim to demonstrate the association between various genotypes of *APOA*1 -75 G/A, +83 C/T and ACS.

## Material and Methods

The current cross-sectional case-control study was conducted at Advanced Center for Human Genetics, Sheri-I-Kashmir Institute of Medical Sciences (SKIMS), North India. A total of 240 subjects were included in the study comprising of 90 ACS patients and 150 healthy controls free from any disease who visited the hospital for general checkups. Consent information was duly sought from each ACS patient and healthy controls. Clinical parameters were recorded according to the given proforma. The inclusion criteria of ACS patients included: ACS with or without undergoing percutaneous coronary intervention, ST elevation myocardial infarction, non-ST elevation myocardial infarction, unstable angina. The exclusion criteria was history of bleeding diathesis, stroke less than 3 months platelet count<70 000/mm^3^, hematocrit <30%

### Extraction of genomic DNA and polymerase chain reaction for amplification

The blood samples of ACS patients and healthy controls were subjected to extraction of genomic DNA by common phenol-chloroform method as well as kit based DNA Extraction (Zymo Research Corporation, USA). The quality and quantity was determined by absorbance at 260nm and 280nm in a Spectrophotometer or by running on 1% agarose gel. The isolated DNA was stored at -20C◦ until for further analysis.

For amplification of *APOA*1-75G/A (rs1799837) and +83C/T (rs5069) polymorphic regions, a 25ul reaction containing genomic DNA 250 ng/mL was used with other ingredients as 1x PCR buffer: 100 mM Tris–HCl, pH 8.3; 500 mM KCl; 15 mM MgCl_2_; deoxyribonucleotide triphosphate (Biotools,B & M Labs, Madrid, Spain): 10mM dATP; 10mM dCTP; 10mM dGTP; 10mM dTTP, primers (Sigma–Aldrich, USA): 10 pM in sterile deionized water and *Taq DNA polymerase* 5 U/μL (Biotools, Madrid, Spain). A single primer pair (Sigma–Aldrich, USA) was designed (using primer3 software) to amplify the required 433bp amplicon covering both SNPs within the *APOA1* region with forward primer 5′-AGGGACAGAGCTGATCCTTGAACTCT TAAG-3ʹ and reverse primer 5ʹ-TTAGGGGACACCTAGCCCTCAGGAAGAGCA-3ʹ (reverse).^16^ The thermal cycling conditions were included as: an initial denaturation at 95°C for 7 min, followed by 35 cycles at 95°C for 35 s, 63°C for 35 s and 74°C for 35 s. The final extension step was at 72°C for 10 minutes. The amplified PCR products were separated by electrophoresis on an agarose gel (1.5%) stained with ethidium bromide. The gel was visualized under ultraviolet light with a 100bp ladder.

### Polymerase chain reaction-restriction fragment length polymorphism (PCR-RFLP)

**Figure 1:**
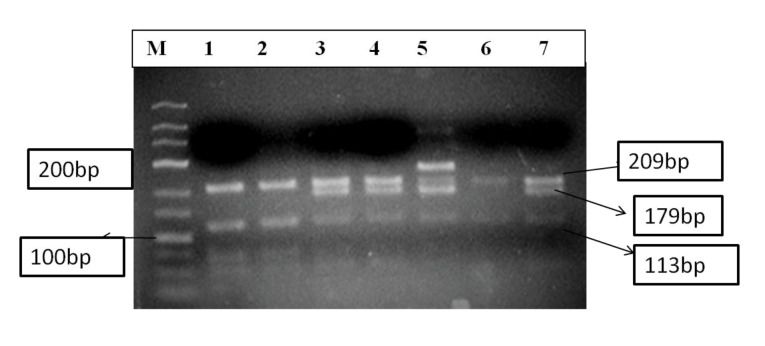
Electrophoresis in a 3 % agarose gel after the MspI digested in CAD cases, lane 1 molecular size marker 25 base pair. Lane 1, 2, 3, 4, 6 GG/CC, lane 5 GA/CT, lane 7 GA/CC

PCR-RFLP was performed to genotype the *APOA*1-75 G/ A and +83 C/T polymorphic sequence variants using 5-10 units of restriction endonuclease enzyme *MspI*(NEB, New England Biolabs), to digest amplified product overnight at 37°C. The digested product was subjected to electrophoresis in a 3% agarose gel followed by ethidium bromide staining and ultraviolet illumination to identify the genotypes. *MspI* restriction site at *APOA*1 -75 bp (G allele) and at +83 bp (C allele) digests into 209, 113, 45 and 66bp. The lack of the restriction site at-75 (A allele) and +83(T allele) displays 3 products of 209, 179, 45bp and one single product 254bp respectively (Figure 1).

For quality control and reproducibility of results, two independent researchers randomly selected 10% samples from both control and cases for genotyping.

### Statistical analysis

IBM Statistics SPSS software (Version-23) was used for statistical evaluation. The cases and controls were compared using the Chi-square test for various categorical parameters, likegender and age of the demographic variables. A goodness-of-fit Chi-square test was used to analyze if the polymorphisms were in Hardy-Weinberg equilibrium between cases and controls. Odds ratios (OR) were used as estimates of the relative risk, and 95% confidence intervals (CI) were calculated to estimate the association between certain genotypes or other related risk factors of ACS.*P* values for all analysis below the level of 0.05 are taken as statistically significant (*P* < 0.05).

## Results

The clinico-pathological characteristics of ACS patients and controls are listed in Table 1. The cases and controls were frequency matched in terms of their age, gender, and smoking status. In this study ratio of the cases, male: female was 3:1 (67 versus 23 respectively).The mean age was 60 ± 15 years for cases and 55 ± 15years for the controls. Of the total number of cases and controls, 61(72.6%) and 115(77%) were smokers respectively. In the study groups, no significant sex-related or smoking status differences were observed between the cases and controls (*P* > 0.05). In cohort group, 81.4% ACS cases had hypertension. ACS STEMI group numbered 71% as compared to 22.2% while as preMI included 19.1% versus 80.9% with no pre MI history. The demographic features of the rest of parameters for ACS patients are recorded in Table 1.

**Table 1:** Characteristics of ACS patients and controls for polymorphic analysis of APOA-gene

**Demographic feature**	**Cases (n=90)**	**Controls (n=150)**	***P*** ** value**
Age	≤55	23(25%)	53(35%)	0.1
	>55	67(75%)	97(65%)	
Gender	Male	67(75%)	123(82%)	0.1
	Female	23(25%)	27(18%)	
Smoking status	Smoker	61(72.6%)	115(77%)	0.3
	Non-smoker	23(27.3%)	35(23%)	
*Hypertension	Hypertensive	66(81.4%)		
	Non-hypertensive	15(18.5%)		
*Diabetes mellitus	Diabetic	33(39.7%)		
	Non-diabetic	50(60.24%)		
*PCI	Yes	62(83.7%)		
	No	12(16.2%)		
*ACS	STEMI	64(71%)		
	NSTEMI	20(22.2%)		
	USA	6(6.66%)		
*Pre-MI	Yes	17(19.1%)		
	No	72(80.9%)		

Abbreviations:PCI, percutaneous coronary intervention; ACS, acute coronary syndrome; STEMI, ST-elevation myocardial infarction; NSTEMI, non-ST elevation myocardial infarction; USA, unstable angina; Pre-MI, previous myocardial infarction * Description of clinico-pathological parameters in cases (patients)

### APOA1 -75 G/A polymorphisms and ACS

In *APOA1* -75 G/A, frequencies of GG, GA and AA genotypes among cases were 49 (55%), 39 (43.33%) and 02(1.66%) while in controls as 79(53%), 62(41%), and 9(6%), respectively. Three different genetic models were performed to see the relation of different genotypes among cases and controls which include additive, dominant and recessive model as shown in Table 2. Overall, the distribution of genotypes for *APOA*1-75G/A among cases and controls was seen almost proportionally the same in all the genetic models performed that showed no significant difference among two groups (*P* > 0.05; Table 2).When classified further into groups, no major difference in frequency of *APOA*1-75G/A genotype was found in age, gender or patients with different smoking status as against the control group (*P* > 0.05; Table 3).

**Table 2:** Overall distribution of genotypes/allele APOA1(-75 and +83)frequencies incases and controls

**Overall Genotyping**	**APOA1 genotype**	**Cases (%)** **N=90**	**Controls (%)** **N=150**	**OR (95% CI)**	***P*** ** value**
APOA1-75G/A (rs1799837)	-75GG	49(55.0)	79(53.0)	Reference	
	-75GA	39(43.3)	62(41.0)	1.0(0.5-1.9)	0.5
	-75AA	2(1.6)	9(6.0)	0.2(0.03-2.3)	0.1
*Dominant Model	GG	49(55.0)	79(53.0)	Reference	0.4
	GA+AA	41(45.0)	71(47.0)	0.9(0.4-1.7)	
**Recessive Model	AA	2(1.6)	9(6.0)	Reference	0.1
	GA+GG	88(98.3)	141(94.0)	3.7(0.4-32.0)	
Additive Model	GG	49(55.0)	79(88.7)	Reference	0.1
	AA	2(1.6)	9(6.0)	0.2(0.03-2.3)	
Allele frequency	-75 G	138(76.6)	220(73.5)	Reference	0.3
	-75 A	42(23.3)	80(26.5)	0.8(0.4-1.4)	
APOA1+83C/T (rs5069)	+83CC	75(83.3)	82(55.0)	Reference	
	+83CT	13(15)	50(33.0)	0.3(0.1-0.6)	0.002
	+83TT	2(1.6)	18(12.0)	0.09(0.01-0.7)	0.004
***Dominant Model	CC	75(83.3)	82(55.0)	Reference	0.0001
	CT+TT	15(16.6)	68(45.0)	0.24(0.1-0.5)	
****Recessive Model	TT	2(1.6)	18(12.0)	Reference	0.01
	CT+CC	88(98.3)	132(88.0)	8.04(1.0-63.5)	
Additive Model	CC	75(97.4)	82(82.0)	Reference	0.004
	TT	2(2.6)	18(18.0)	0.09(0.01-0.7)	
Allele frequency	+83 C	163(90.5)	214(71.5)	Reference	0.0001
	+83 T	17(9.4)	86(28.5)	0.25(0.1-0.5)	

Abbreviations:GG: wild; GA: heterozygous; AA: homozygous variant. CC: wild; CT: heterozygous; TT: homozygous variant. * Dominant model: GG, ** Recessive model: AA, ***Dominant model: CC, **** Recessive model: TT

**Table 3:** Clinical-epidemiological variables of CAD patients versus the polymorphic phenotypes of the APO-A1 gene

**Parameter**	*Cases APOA1 -75 G/A		**Control**		**OR(95%** **C.I)**	***P*** **value**	**Cases APO-A1 +83 C/T		**Control**		**OR(95% C.I)**	***P*** ** value**
		**GG (%)**	**GA+** **AA (%)**	**GG (%)**			**GA+** **AA (%)**	**CC (%)**	**CT+** **TT (%)**	**CC (%)**			**CT+** **TT (%)**
Age	≤55	12(24.23	11(25.9)	24(30)	44(50)	0.4(0.1-1.5)	0.1	20(26)	3(20)	38(40)	15(55.5)	0.3(0.07-2)	0.2
	>55	7.5(75.7)	30(74)	56(69.8)	42(50)	1.0(0.4-2.2)	0.5	55(74)	12(80)	55(59.6)	12(44.5)	0.4(0.17-1.2)	0.06
Gender	Male	42(69.6)	33(81.5)	57(71.6)	54(76.6)	1.0(0.48-2.1)	0.5	57(76)	10(70)	61(74.5)	58(86.6)	0.19(0.07-.48)	0.00
	Female	15(30)	8(18.5)	23(28.3)	17(23.2)	0.7(0.24-2.0)	0.5	18(24)	5(30)	22(25.4)	10(13.3)	0.6(.18-2.11)	0.3
Smoking Status	Smoker	34(69.7)	27(78.3)	63(79.2)	58(82.9)	0.84(0.4-1.8)	0.4	55(81)	6(40)	67(81.8)	52(22.8)	0.14(0.04-.44)	0.00
	Non-Smoker	16(30.3)	8(21.7)	17(20.7)	13(17)	0.69(0.2-2.8)	0.4	14(19)	9(60)	16(18)	172(77)	0.06(0.01-.19)	0.00
HTN	Yes	40(84.4)	25(77.3)	-	-	0.6 (0.15-2.5)	0.3	55(82)	10(78)	-	-	0.74(0.13-.22)	0.5
	No	8(15.6)	8(22.7)	-	-			13(17.7)	4(22)	-	-		
DM	Yes	17(33.3)	16(50)	-	-	2(0.67-6.04)	0.1	24(34.8)	9(64.2)	-	-	3.75(0.83-17)	0.07
	No	33(66.6)	17(50)	-	-			45(65.2)	5(35.7)	-	-		
PCI	Yes	36(88.8)	25(77.3)	-	-	0.4(0.08-2.0)	0.2	49(83)	12(80)	-	-	0.73(0.12-4.3)	0.5
	No	5(11)	8(22.7)	-	-			10(17)	3(20)	-	-		
ACS	NSTEMI	10(20.8)	9(21.4)	-	-	Ref		15(21.2)	12(60)	-	-		
	STEMI	33(68.7)	31(73.8)	-	-	1.1(0.3-3.8)	0.5	52(74.4)	5(25)	-	-	0.7(0.1-3.4)	0.4
	USA	5(10.41)	2(4.7)	-	-	2.8(0.2-29.7)	0.3	4(4.2)	3(15)	-	-	0.2(0.02-1.8)	0.1
PREMI	Yes	12(16)	7(17.07)	-	-	0.44(0.15-1.2**)**	0.1	35(46)	5(35.7)	-	-	0.5(0.13-2.6)	0.3
	No	63(84**)**	34(82.9)	-	-			40(54)	9(64.2)	-	-		

Abbreviations: HTN, hypertension; DM, diabetes mellitus; PCI, percutaneous coronary intervention; STEMI, ST-elevation myocardial infarction; NSTEMI, non-ST elevation myocardial infarction; USA, unstable angina; pre-MI, previous myocardial infraction *Association of APOA1 -75 G/A wild type (GG**)** and variant genotype (GA+AA) in demographic/pathological features between cases and controls. ** Association of APOA1 +83 C/T wild type (GG**)** and variant genotype (CT+TT) in demographic/pathological features between cases and controls

When the patients were classified with respect to different pathologies, like hypertension, although variant genotype GA+AA was found more often in hypertensive (77.3%) than non-hypertensive (22.7%) group, yet the difference was insignificant, OR = 0.63 (C.I:0.15-2.5; *P=0.3*). Despite the differences in the frequency of *APOA*1-75G/A genotypes among different parameters of ACS, none of them could achieve the significant association (*P* > 0.05) (Table 3).

### APOA1 +83 C/T polymorphisms and ACS

In *APOA*1 +83 C/T, frequencies of CC, CT and TT genotypes observed among cases were 75 (83.33%), 13 (15%) and 02 (1.66%), while in healthy controls frequencies were 82(55%), 50(33%) and 18(12%) respectively (Table 2). The distribution of APOA1 +83 C/T homozygous TT and heterozygous CT genotypes were significantly more among cases than controls as 1.6% vs. 12% for TT (*P* = 0.004) and 15% vs. 33% CT (*P=0.002*). In case of dominant model, combined genotype CT+TT was found more often in controls 45% than cases 16.6%, with an OR = 0.24 (C.I:0.11-0.53;* P=*0.0001). The frequency of variant ‘T’ allele observed in cases and controls was found to be 9.4% versus 28.5% respectively with an OR=0.2 (C.I:0.12-0.50; *P*= 0.00001). In males, CT+TT genotype was found higher (76.6%) in controls than 81.5% in cases (*P=*0.0001) while as the rest of parameters did not differ as shown in Table 3.

When the patients were classified as cohort with respect to different pathologies as shown in Table 3, no significant differences were observed within different parameters with respect to variant genotypes of *APOA*1 +83 C/T.

Haplotypic analysis was performed to demonstrate the pattern of linkage disequilibrium and few haplotypes were found with frequencies more than 5% among both cases and controls and those with <1% frequency was not considered. Table 4 shows the frequencies for the estimated 2-marker haplotypes among patients and controls. The haplotype observed in cases and controls with highest frequency as G/C haplotype that accounted for 40.0% in cases and 27.4% in controls of the SNPs studied haplotypes (*APOA*1-75G/A and*APOA*1 +83 C/T). Haplotype GT showed a marked difference when compared with wild type GC between cases and controls (*P* < 0.0001) as depicted in Table 4.

**Table 4:** Haplotypes analysis of APOA1 -75 G/A +83 C/T for overall association with ACS cases and healthy controls

*Haplotypes **APOA1 -75 APOA1+83**	**Controls** **150(%)**	**Cases** **90(%)**	**OR (95%CI)**	***P*** ** value**
G	C	82(27.4)	72(40)	1(Ref)	
G	T	28(9.4)	4(2.2)	7.9 (2.6-23.8)	<0.0001
A	C	20(3.4)	4(2.2)	0.4(0.08-2.5)	0.4
A	T	82(27.4)	0	-	-

*The two SNPS on the same chromosome were analyzed for haplotype analysis to demonstrate the cumulative impact on ACS

## Discussion

Apolipoprotein A1, a protein in humans is encoded by the *APOA*1 gene that has a vital role in lipid metabolism. *APOA*1 is a major protein ingredient of HDL and a relatively abundant plasma protein.^17^ In the *APOA*1 gene -75 G/A and +83C/T, play an important role in lipid metabolism.^18-20^ Regarding ACS, the studies have been very limited to reach the conclusive remark. A cross sectional case-control study was conducted to observe the role of *APOA*1 gene -75 G/A and +83C/T variations with respect to ACS in our population (Kashmir province, North India). In our report, *APOA*1 -75 G/A, the frequency of three genotypes of *APOA*1-75 G/A SNP among ACS patients and the controls revealed no significant differences. Similar scenario has been published by Yan Ding et al,^21^ that reported lack of association for *APOA*1-75 G/A in ACS. Further, *APOA*1 -75 G/A and ACS risk was not proved in European population with 98.6%, 1.3% and 0.1% genotypic frequency in cases and 99.2%, 0.8% and 0% in controls.^22^ It is worthwhile to mention that the frequency of *APOA*1-75 G/A varies considerably among different ethnic regions as can be seen among three studies which show quite different genotypic differences in both cases and controls. In our study frequency of variant A allele *APOA*1 -75 G/A showed no association which is supported by similar scenario reported by Yan et al.^21^ On the contrary, a study in Australian population, ^18,19^ found that the presence of the variant A allele increased the severity of ACS, a finding that is in stark contrast to our study and one more report carried earlier.^21^

In this study, combined genotype (GA+AA) was found comparatively higher in smokers of control group (controls: 82.9% versus cases 78.3%). This is in discordance with the study by Morgan et al.^23^ Also, the combined genotype (GA+AA) was found more often in hypertensive in accordance with the study performed in the Chinese population.^13^ Interestingly, in contrast to our study, Morgan et al,^23^ has shown an association between the *APOA*1-75G/A polymorphism and diabetes mellitus (DM). The possible reasons for this contradiction are unclear though these could be due to different geographical regions. It must be stressed that even among populations of similar ethnicity and gene pool, environmental factors such as diet, stress and physical inactivity compound to an individual’s composite risk for ACS in inexplicable proportions.

In our study, *APOA*1 +83C/T showed a protective role with respect to the development of ACS where the frequencies of different genotypes showed significant differences between cases and controls. The frequency of homozygous variant genotype TT allele was observed significantly higher in controls than cases (12% versus 1.6% respectively). This clearly demonstrates a significant association of *APOA*1+83 C/T variant genotype with ACS in our population. *APOA*1 +83 variant T allele was found to be higher in controls (0.34 versus 0.12) than cases and clearly demonstrates that T allele has uniquely a protective role for ACS in our population. In contrast to our findings, Morgan et al,^23^ found no association between ACS and *APOA*1 +83 C/T gene polymorphism. The proposed protective role of *APOA*1 +83 variant T allele as found in our study is substantiated by an investigation by Wang et al. who found T allele associated with increased levels of HDL and *APOA*1, an evidence that suggests protective role over ACS.^19^ One of the possible explanations for this transition is that *APOA*1 +83 reside in the 5ʹ region of the *APOA*1 gene. The *MspI* site at *APOA*1+83bp embedded with CpG island is known to be hypermethylated in non-expressing cells but demethylated in cells expressing *APOA*1. It is possible that T substitution at this site may lead to further demethylation, resulting in more cells expressing the gene.

Further, in our study *APOA*1 +83 C/T variations was associated with ACS in men. This is partially in accordance with an earlier study that found no association in men and women.^23^

Our study found no significant association of ACS was found with hypertension and diabetes. Our results are in accordance with another study conducted earlier.^23^ On the basis of cardiovascular events pre-MI, STEMI, NSTEMI and USA, no association with *APOA*1 +83 C/T was found in contradiction with the study by Morgan et al.^23^ Discrepancies in the findings of these studies with our report could be attributed to differences in the genetic susceptibility between different ethnic groups. Also, the *APOA*1 gene locus resides in a cluster with CIII and AIV loci, which could attribute to linkage disequilibrium with different *APOA*1 alleles in some but not in other populations.

The putative role of *APOA*1 -75 G/A and +83 C/T has been studied in various diseases across the globe where prominent diseases that have been complied in this study to highlight the implication and role of this sequence variation include hypertension,^24^ renal cancer,^15^ Alzheimer’s disease,^22^ CAD,^13^ and all the diseases have shown distinct but varied associations. This augments for the need to study the role of *APOA*1 in more diseases.

Further, the effect of a single polymorphic nucleotide variation is questionable to lead to an outcome in the study of multifaceted diseases. Therefore, the amalgamation of different genetic variants in the set of connections of same loci strengthens their efficiency to improve the analytical influence for complex diseases.^25,26^ To demonstrate whether *APOA*1−75 G/A and +83 C/T polymorphic variants could translate a synergistic effect in the development or progression of ACS, we looked at their collective impact to examine the inclination to the disease. The most frequently implicated haplotype was GT variant haplotype that showed a significant protective impact on ACS as compared to wild type haplotype GC. Our study showed the haplotypes at two restriction sites as more informative and demonstrated a significant association together to confer a protective role in ACS. In consistence with our data, there a few reports that have shown more or less the same trend,^27,28^ implicating that combining both polymorphic sites of *APOA*1 −75 G/A and +83 C/T sites as more informative and significantly portray the combined effect with almost twice the amount of phenotypic variation in plasma *APOA*1compared to single RFLP site. This study shows that haplotype analysis in the *APOA*l gene is very important to display its functional significance in ACS.

## Conclusion

The study concludes that *APOA*1 -75G/A has no association with ACS but its other related polymorphic variant T allele and TT genotype of +83 C/T was observed to confer a protective effect with respect to the same condition. Our report further implies that a particular haplotype (GT) of *APOA*1 gene is highly implicated and possibly can act as low penetrance genotypes in the predilection to ACS. These findings are subject to be validated in large sample cohort studies to decide the course of ACS management at molecular perspective.

## Acknowledgments

The authors acknowledge the Prof Lily Want (Head, Department of English, University of Kashmir, India) for her generous help to edit the manuscript. The authors further acknowledge the technical staff of Department of cardiology, SKIMS, Srinagar, for their timely help in procuring blood samples and patients for giving their consent to participate in the study.

## Competing interest

All authors declare no competing interest.

## Ethical approval

The procedures done in the study involving human participants were in agreement with the ethical standards of the institutional and/ or national research committee and with the 1964 Helsinki declaration and its later amendments or comparable ethical standards. Ethical approval for this study was obtained from Institutional Ethical Review Committee (IEC SKIMS Study ref: 1904/2014)

## Funding

None.
